# Bridging the Gap: Transportation, Aging, and Access to Healthcare

**DOI:** 10.1093/geroni/igaf122.2482

**Published:** 2025-12-31

**Authors:** Zeve Marcus, Katherine Freund

**Affiliations:** ITNAmerica, Westbrook, Maine, United States; ITNAmerica, Westbrook, Maine, United States

## Abstract

ITNAmerica, the first national nonprofit transportation network for older adults and people with mobility challenges, is dedicated to promoting lifelong mobility. By integrating a 178-field research database into ITNRides’ transportation technology, ITNAmerica has built a 1.6 million ride database to provide critical insights into how mobility influences the social determinants of health. With support from the CareQuest Institute for Oral Health, ITNAmerica is conducting a multi-phase research initiative to examine how transportation impacts healthcare access, particularly oral healthcare, for older adults. This project is in three phases: 1) migrating 1.6 million rides from 26 legacy databases into a unified, multi-tenant database on the Salesforce platform to improve data integrity and accessibility; 2) conducting a detailed statistical analysis of these rides to identify transportation patterns and disparities; and 3) planning a longitudinal study to assess the long-term impact of transportation on health outcomes. Transportation is vital for older adults to access healthcare, social engagement, and daily activities, yet barriers such as limited mobility, geographic isolation, and affordability contribute to disparities, particularly among low-income, rural, and minority populations. By leveraging decades of transportation data and engaging both a Scientific Advisory Council and a Diversity Panel, this initiative ensures that research findings reflect the needs of underserved communities while generating actionable insights to inform policies and programs that promote health equity. This project provides a unique opportunity to understand and address transportation as a critical factor in healthy aging.

